# Supplementary material for: Scaffold-mediated gating of Cdc42 signalling flux
Source: eLife. 2017 Mar 17;6:e25257. doi: 10.7554/eLife.25257 (PMC5386590; doi:10.7554/eLife.25257)
Supplement: Supplementary file 1. — DOI: http://dx.doi.org/10.7554/eLife.25257.014 [file elife-25257-supp1.docx]

| Name | **Genotype** | **Reference** |
| --- | --- | --- |
| DMY349 | *MATa, his3-11, leu2-3,112, trp1-1, ura3-52, ade2-1, can1- 100, GAL+, bar1* | (Altman and Kellogg, 1997) |
| DMY570 | *MATa, his3-11, leu2-3,112, trp1-1, ura3-52, ade2-1, can1- 100, GAL1-CDC24::kanMX6, bar1* | This study |
| DMY939 | *MATa, his3^0, leu2^0, trp1^63, ura3^0, GAL+* | Grant Hartzog lab. |
| DMY2000 | *MATa, his3^1, leu2^0, met15^0, ura3^0, SSD1-V, bar1Δ::kanMX6, cdc42Δ::CaURA,* Containing pDM303 (pRS315 *mEOS-CDC42*)*.* | This study |
| DMY2023 | *MATa, his3^1, leu2^0, met15^0, ura3^0, SSD1-V, bem1Δ::kanMX6, cdc42Δ::CaURA3,* Containing pDM303 (pRS315 *mEOS-CDC42*)*.* | This study |
| DMY2033  (*Pichia pastoris, X-33*) | Mut+ Containing pDM401 (pPICZ-*6xHis-StrepTagII-CDC42*) | This study |
| DMY2147 | *MATa, his3-11, leu2-3,112, trp1-1, ura3-52, ade2-1, can1- 100, GAL+, CDC24-3xHA::URA3, bar1* | This study |
| DMY2151 | *MATa, his3-11, leu2-3,112, trp1-1, ura3-52, ade2-1, can1- 100, GAL+, cdc24-46A-3xHA::URA3, bar1* | This study |
| DMY2154 | *MATa, his3-11, leu2-3,112, trp1-1, ura3-52, ade2-1, can1- 100, GAL+, cdc24-28D-3xHA::URA3, bar1* | This study |
| DMY2249 | *MATa, his3-11, leu2-3,112, trp1-1, ura3-52, ade2-1, can1- 100, GAL+, CDC24-3xHA::URA3, rga1Δ::HIS5, bar1* | This study |
| DMY2251 | *MATa, his3-11, leu2-3,112, trp1-1, ura3-52, ade2-1, can1- 100, GAL+, cdc24-46A-3xHA::URA3, rga1Δ::HIS5, bar1* | This study |
| DMY2253 | *MATa, his3-11, leu2-3,112, trp1-1, ura3-52, ade2-1, can1- 100, GAL+, cdc24-28D-3xHA::URA3, rga1Δ::HIS5, bar1* | This study |
| DMY2297 | *MATa, his3-11, leu2-3, 112, trp1-1, ura3-52, GAL*+, *cdc24- 46A-3xHA::URA3, bem1Δ::kanMX6* | This study |
| DMY2300 | *MATa, his3-11, leu2-3, 112, trp1-1, ura3-52, GAL+, Cdc24- 3xHA::URA3, bem1Δ::kanMX6* | This study |
| DMY2303 | *MATa, his3-11, leu2-3, 112, trp1-1, ura3-52, GAL+, cdc24- 28D-3xHA::URA3, bem1Δ::kanMX6* | This study |
| DMY2333 | *MATa, his3-11, leu2-3,112, trp1-1, ura3-52, ade2-1, can1- 100, GAL+, cdc24-15A-3xHA::URA3, bar1* | This study |
| DMY2334 | *MATa, his3-11, leu2-3,112, trp1-1, ura3-52, ade2-1, can1- 100, GAL+, cdc24-15D-3xHA::URA3, bar1* | This study |

DMY570 was transformed with pDM700 (pRS416 *pCYC1-CDC24-mEOS*), pDM701 (pRS416 *pCYC1-cdc24-46A-mEOS*) and pDM704 (pRS416 *pCYC1-cdc24-28D-*

*mEOS*) plasmids for imaging Cdc24 *in vivo*.

Altman, R., and Kellogg, D. (1997). Control of mitotic events by Nap1 and the Gin4 kinase. J Cell Biol

*138*, 119-130.
